# Modern Sociology

**Published:** 1901-04-20

**Authors:** 


### MODERN SOCIOLOGY.

### DANGEROUS TRADES.

### LEAD POISONING IN POTTERIES.

THE danger attending workers in potteries through handling lead in one form or another has attracted the attention of those in authority much in late years, and Professors Thorpe, Principal of the Government Laboratory, and Oliver, of the Royal Infirmary, Newcastle-on-Tyne, were entrusted by the Government with the investigation of the question. Their report was published in 1899, and though it clearly shows the dangers that surround pottery work, it also shows that now these dangers are appreciated the majority of master potters are doing their best to lessen them and, where possible, to do away with them altogether.

Lead is an ingredient in almost all existing glazes. White lead is generally used, and one of the matters into which the gentlemen of the committee were to inquire was whether some less soluble compound of lead might not be substituted for this, or, better still, if a leadless glaze could not be employed. These points the commissioners were able to answer satisfactorily. Experienced manufacturers, speaking with full consideration of profit as well as safety, assured them that no practical difficulties would result from the substitution of what they call "fritted" lead for "raw" lead. Apart from the hygienic question it has even a commercial advantage. "Glazes in which the whole of the lead has been fritted as a proper lead silicate—that is, fritted directly with the other components of the glaze so as to form a double silicate—have been found to possess greater covering power than a glaze containing the same relative amount of lead in a raw state, with the further advantage of enhancing the colour." But much depends on the nature and composition of the "fritted" lead. Some substances which pass under that name, though better than the ordinary raw lead, are not unattended with danger to those who work with it. Indeed, some silicates, which contain about 70 per cent. of lead oxide, though known as "fritted" lead, are hardly less soluble in acids than the ordinary basic lead carbonate. It is possible, however, to make a glaze which shall contain only 22 per cent. of lead monoxide, and which is only very slightly attacked by even strong hydro-chloric acid. In contrast with this, which any manufacturer could use without inconvenience, some potters use glazes where lead is employed "indiscriminately and even recklessly." "We have found," say the commissioners, "glazed ware so heavily leaded that vinegar and the acids of fruit will in a few hours extract a very sensible quantity of the metal."

A difficulty in making any regulation as to the amount of lead to be used in glaze arises from the fact that manufacturers themselves seem to have no fixed rule as to the necessary quantity. Some say that if the glaze contains 10 per cent. of lead it is sufficient, while others place the minimum as high as 20 per cent. In samples taken from various dipping-tubs, the Commissioners found the amount of lead vary from 13 to 24 per cent., but as they found excellent lead-glazed ware in which the amount of lead did not exceed 12 per cent. of the weight of the materials, they recommend that "in the interest of the public as well as that of the worker," the amount of "fritted" lead in the dipping tub, calculated as lead monoxide, should not exceed 12 per cent. of the dried materials. If this limit received legislative sanction, the factory inspectors should be empowered to take samples from the dipping-tubs in the factories they visited, to see if the legal limit were being exceeded.

Considerable difficulty arises about the employment of women in potteries. On the one hand, the operation of dipping the ware in the glaze, and of cleaning it after dipping, is light work, suitable to women's strength. Women are also employed in painting the ware, both in the decorating of majolica tiles and earthenware, and in applying enamel colours for ground-laying, colour-dusting, and litho-transfers. On the face of it, all these occupations seem to be particularly suited for women, but in all they are working with materials in which lead is prominently present, and it has been found that young persons and women between the ages of 17 and 30 are peculiarly liable to lead-poisoning. In some potteries on the Continent protection is afforded—at least to the dippers—by their wearing india-rubber gloves which extend up to the elbows, and largely prevent the skin absorbing any lead. These gloves are not popular here. It is alleged that the gloves are soon cut to pieces by the biscuit, and that their use prevents the dipper from modifying the manner of dipping, depending on whether the ware is "hard" or "easy fired," which is judged by the "feel" of the biscuit in the hand. But the one worker whom the commissioners saw wearing gloves was well satisfied with them, and they think that they might be more frequently used with good effect. They are of opinion, however, that young persons and women between the ages of 17 and 30 should not be employed as dippers or their assistants, ware-cleaners after dippers, and glost-placers in factories where lead is used. Happily, however, there is hope that before long lead may be entirely excluded from the dipping-tub. Already all plain ware can be glazed with leadless glaze. As the report says: "We have no doubt whatever that leadless glazes of sufficient brilliancy, covering power, and durability, and adapted to all kinds of table, domestic, and sanitary ware, are now within the reach of the manufacturer." The words are italicised. Leadless glazes can be used for white, cream, buff, and printed tiles, for finger-plates, door-knobs, and all the fittings known in the trade as "china furniture," and also for insulators, and other bits of china required for the application of electricity to domestic uses. Thus all necessary earthenware may be made without risking the health of the worker, and even if private persons, with their ideas of taste, may not be ready to confine themselves to such plain ware, it would be a great help if public bodies and institutions, schools, hospitals, workhouses, Government offices, and the like, demanded that all the pottery they need shall be made with leadless glaze. The Royal Worcester Pottery Works have also announced lately that they have succeeded in glazing highly-decorated china with leadless glaze without any loss of brilliancy in the colouring.

This leaves only the paintresses of various kinds to be seen to, and it is to be regretted that the most of the colours they use contain from 15 to 50 per cent. of lead, though this is usually "fritted." For them the commissioners recommend that fans should be used to carry away as much as possible the impalpable dust in which the colour is apt to float around them. They should also be subject to frequent medical examination, should have overalls and abundant washing conveniences provided, and should be strictly prohibited from eating or drinking in the workshops. It is important also that the workers should become so far alive to the dangers they run that they will avail themselves oft he conveniences provided for them. Perhaps this is more difficult to attain than anything else.

